# Supplementary material for: Validation of plasma microRNAs as biomarkers in sepsis associated acute kidney injury upon first clinical presentation reveals limited diagnostic and prognostic performance
Source: PLoS One. 2025 Sep 4;20(9):e0331442. doi: 10.1371/journal.pone.0331442 (PMC12410816; doi:10.1371/journal.pone.0331442)
Supplement: S3 Fig — Overview of rate of missing microRNA values per cohort. (DOCX) [file pone.0331442.s006.docx]

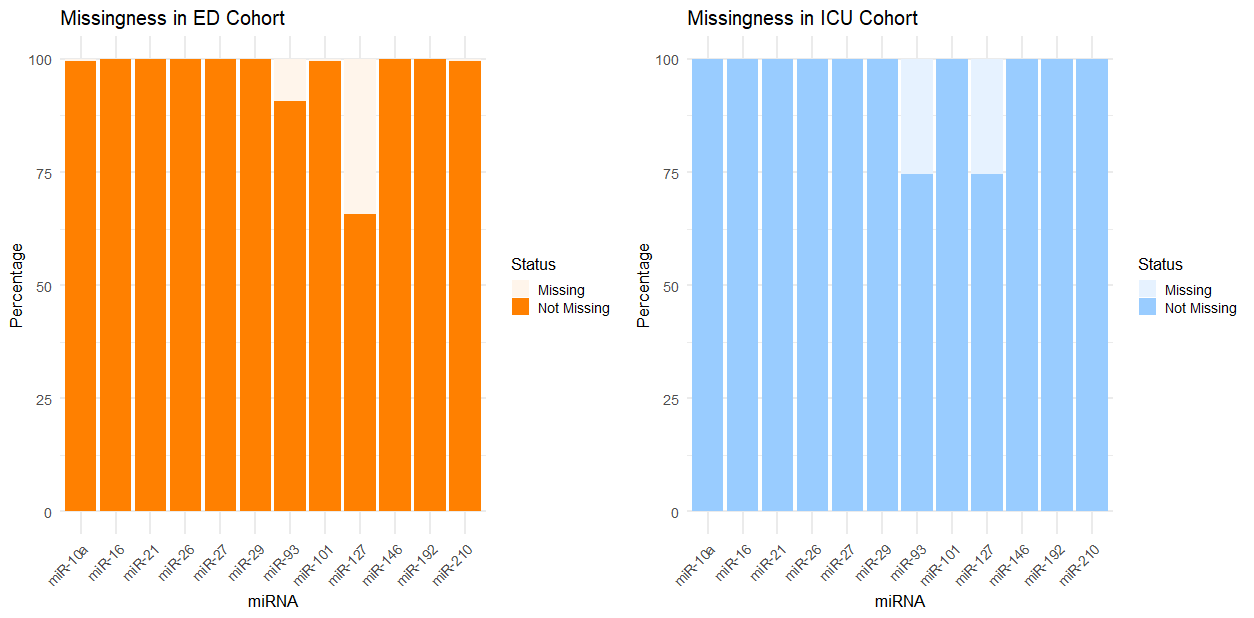


| miRNA | **ED cohort** | **ICU cohort** |
| --- | --- | --- |
| miR-10a | 1 (<1) | 0 |
| miR-16 | 0 | 0 |
| miR-21 | 0 | 0 |
| miR-26a | 0 | 0 |
| miR-27b | 0 | 0 |
| miR-29b | 0 | 0 |
| miR-93 | 18 (9) | 12 (26) |
| miR-101 | 1 (<1) | 0 |
| miR-127 | 66 (34) | 12 (26) |
| miR-146 | 0 | 0 |
| miR-192 | 0 | 0 |
| miR-210 | 1 (<1) | 0 |

**S3 Figure. Missingness per microRNA and cohort.** Overview of rate of missing microRNA values per cohort.
