# Supplementary material for: Validation of plasma microRNAs as biomarkers in sepsis associated acute kidney injury upon first clinical presentation reveals limited diagnostic and prognostic performance
Source: PLoS One. 2025 Sep 4;20(9):e0331442. doi: 10.1371/journal.pone.0331442 (PMC12410816; doi:10.1371/journal.pone.0331442)
Supplement: S4 Table — Overview of the infection sites observed at Emergency Department (ED) and Intensive Care Unit (ICU) admission across the four subgroups with an infection. (DOCX) [file pone.0331442.s007.docx]

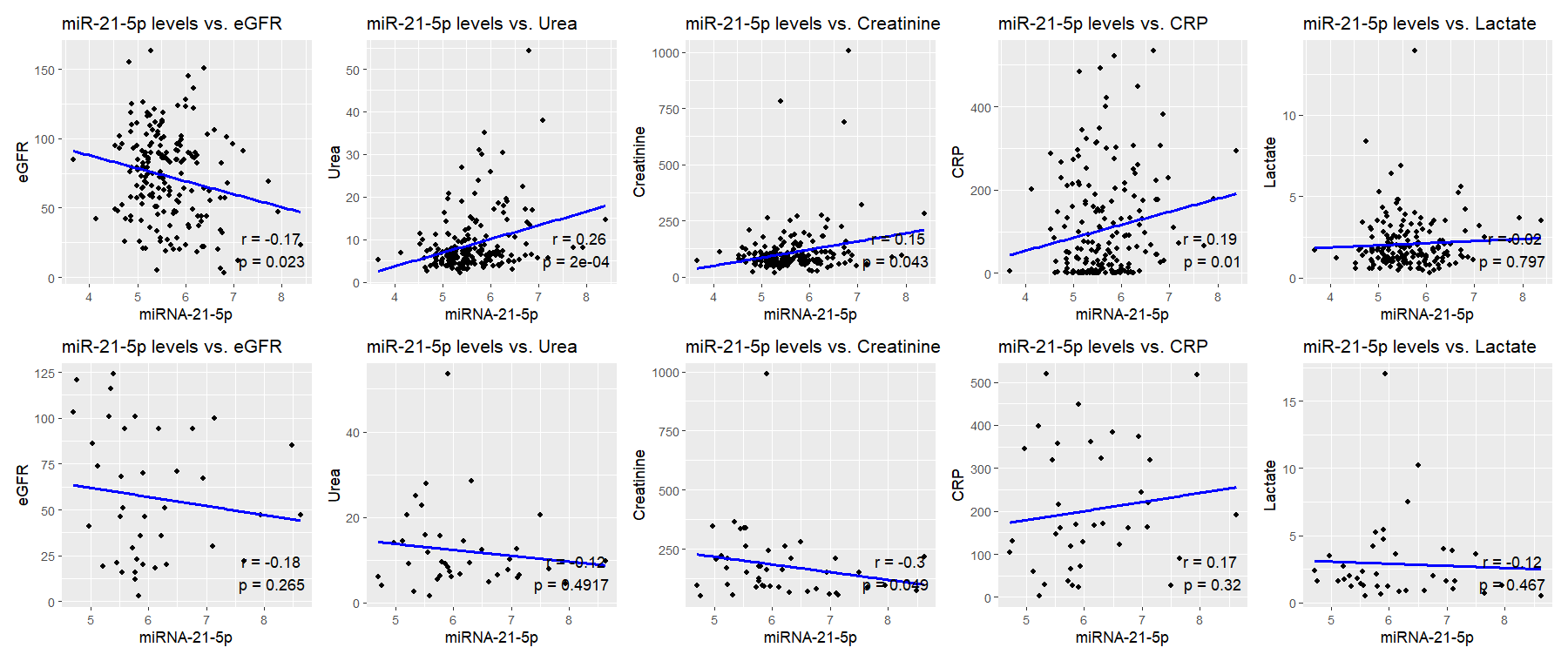


**S4 Figure.** **Scatterplots on correlation laboratory parameters with miR-21-5p.** Scatterplots exploring the correlation of miR-21-5p with laboratory parameters including eGFR, urea, creatinine, CRP and lactate. The upper row represents the ED cohort, while the lower row represents the ICU cohort. Correlation coefficients (r) and corresponding p values are shown.
