# Supplementary material for: Validation of plasma microRNAs as biomarkers in sepsis associated acute kidney injury upon first clinical presentation reveals limited diagnostic and prognostic performance
Source: PLoS One. 2025 Sep 4;20(9):e0331442. doi: 10.1371/journal.pone.0331442 (PMC12410816; doi:10.1371/journal.pone.0331442)
Supplement: S5 Table — Association of circulating microRNA levels with 30-day mortality in the ED cohort. Odds ratio (OR), and area under the receiver operator curve (AUROC) with their respective 95% confidence intervals (CI) are presented in the table below. Bold indicates significant association. (DOCX) [file pone.0331442.s008.docx]

**S5 Table. 30-day mortality associations in the ED cohort.** Association of circulating microRNA levels with 30-day mortality in the ED cohort. Odds ratio (OR), and area under the receiver operator curve (AUROC) with their respective 95% confidence intervals (CI) are presented in the table below. **Bold** indicates significant association.

| microRNA | OR (95% CI) | AUC (95% CI) |
| --- | --- | --- |
| miR-10a-5p | 1.00 (0.72-1.37) | 0.54 (0.41-0.66) |
| **miR-16-5p** | **0.70 (0.51-0.96)** | **0.60 (0.47-0.74)** |
| **miR-21-5p** | **4.26 (2.16-8.38)** | **0.76 (0.65-0.87)** |
| miR-26b-5p | 0.80 (0.53-1.19) | 0.55 (0.42-0.69) |
| miR-27a-5p | 0.98 (0.68-1.41) | 0.50 (0.38-0.61) |
| miR-29a-5p | 1.38 (0.79-2.39) | 0.44 (0.31-0.57) |
| miR-93-3p | 0.97 (0.72-1.30) | 0.53 (0.41-0.65) |
| miR-101-3p | 0.72 (0.46-1.13) | 0.58 (0.45-0.71) |
| miR-127-3p | 1.04 (0.84-1.30) | 0.52 (0.35-0.68) |
| miR-146a-5p | 1.19 (0.86-1.63) | 0.56 (0.44-0.68) |
| miR-192-5p | 1.19 (0.84-1.70) | 0.56 0.43-0.69) |
| miR-210-3p | 1.27 (0.89-1.83) | 0.55 (0.42-0.68) |
