# Supplementary material for: Validation of plasma microRNAs as biomarkers in sepsis associated acute kidney injury upon first clinical presentation reveals limited diagnostic and prognostic performance
Source: PLoS One. 2025 Sep 4;20(9):e0331442. doi: 10.1371/journal.pone.0331442 (PMC12410816; doi:10.1371/journal.pone.0331442)
Supplement: S6 Table — Association of circulating microRNA levels from plasma with 30-day mortality in the ICU cohort. Odds ratio (OR), and area under the receiver operator curve (AUROC) with their respective 95% confidence intervals (CI) are presented in the table below. Bold indicates significant association. (DOCX) [file pone.0331442.s009.docx]

**S6 Table.** **30-day mortality associations in the ICU cohort.** Association of circulating microRNA levels from plasma with 30-day mortality in the ICU cohort. Odds ratio (OR), and area under the receiver operator curve (AUROC) with their respective 95% confidence intervals (CI) are presented in the table below. **Bold** indicates significant association.

| microRNA | OR (95% CI) | AUC (95% CI) |
| --- | --- | --- |
| miR-10a-5p | 0.91 (0.55-1.51) | 0.52 (0.35-0.70) |
| miR-16-5p | 1.20 (0.73-1.96) | 0.59 (0.42-0.75) |
| **miR-21-5p** | **3.34 (1.70-6.55)** | **0.58 (0.41-0.75)** |
| miR-26b-5p | 1.40 (0.68-2.88) | 0.59 (0.42-0.75) |
| miR-27a-5p | 0.82 (0.37-1.80) | 0.54 (0.37-0.71) |
| miR-29a-5p | 1.21 (0.65-2.28) | 0.44 (0.27-0.61) |
| miR-93-3p | 0.79 (0.51-1.22) | 0.63 (0.43-0.83) |
| miR-101-3p | 1.04 (0.44-2.43) | 0.52 (0.35-0.69) |
| miR-127-3p | 0.97 (0.71-1.34) | 0.52 (0.30-0.74) |
| miR-146a-5p | 0.67 (0.34-1.32) | 0.62 (0.45-0.78) |
| miR-192-5p | 0.95 (0.64-1.41) | 0.53 (0.36-0.71) |
| miR-210-3p | 1.24 (0.80-1.94) | 0.60 (0.43-0.76) |
