# Supplementary material for: Validation of plasma microRNAs as biomarkers in sepsis associated acute kidney injury upon first clinical presentation reveals limited diagnostic and prognostic performance
Source: PLoS One. 2025 Sep 4;20(9):e0331442. doi: 10.1371/journal.pone.0331442 (PMC12410816; doi:10.1371/journal.pone.0331442)
Supplement: S5 Fig — Correlation plot including microRNAs, laboratory parameters of inflammation, sepsis and kidney function and acute kidney injury severity based on KDIGO criteria. eGFR – estimated glomerular filtration rate, CRP – C reactive protein, AKI – Acute Kidney Injury, KDIGO – kidney disease improving global outcome. Red indicated a more negative correlation, blue indicates a more positive correlation. Heatmap shows the results from the ED cohort. (DOCX) [file pone.0331442.s011.docx]

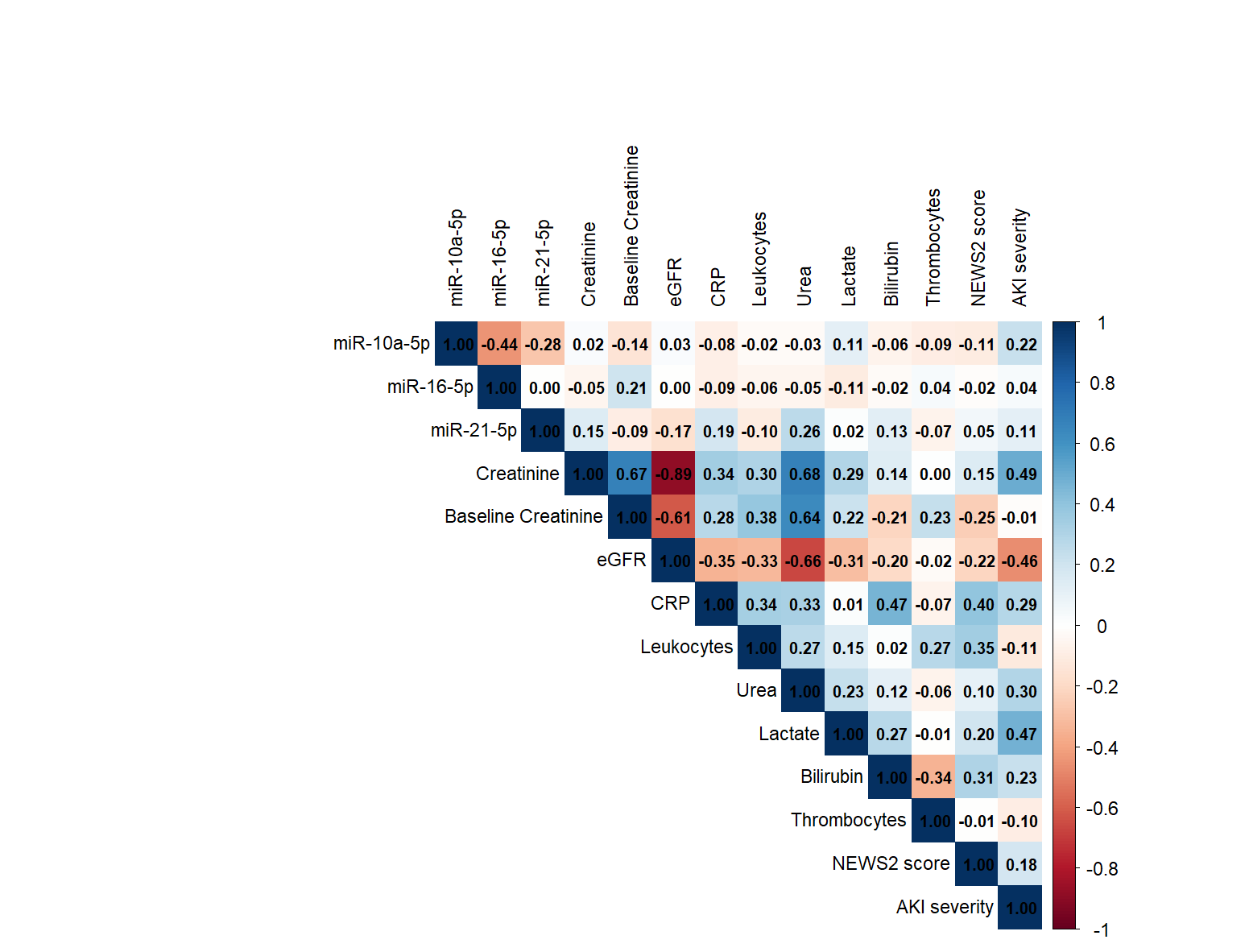


**S5 Figure. Correlation plot microRNAs and clinical parameters.** Correlation plot including microRNAs, laboratory parameters of inflammation, sepsis and kidney function and acute kidney injury severity based on KDIGO criteria. eGFR – estimated glomerular filtration rate, CRP – C reactive protein, AKI – Acute Kidney Injury, KDIGO – kidney disease improving global outcome. Red indicated a more negative correlation, blue indicates a more positive correlation. Heatmap shows the results from the ED cohort.
