# Supplementary material for: Validation of plasma microRNAs as biomarkers in sepsis associated acute kidney injury upon first clinical presentation reveals limited diagnostic and prognostic performance
Source: PLoS One. 2025 Sep 4;20(9):e0331442. doi: 10.1371/journal.pone.0331442 (PMC12410816; doi:10.1371/journal.pone.0331442)
Supplement: S7 Table — Effect sizes are reported as Cohens f for comparison of 3 groups (ED) and Cohens d for comparison of two groups (ICU). For better interpretation of non-significant findings 95% confidence intervals (CIs) are included to illustrate the range of plausible effect sizes. The estimated sample sizes required to achieve 80% power are shown in the last column. (DOCX) [file pone.0331442.s012.docx]

**S7 Table. Post hoc power analysis for the measured miRNA level differences.** Effect sizes are reported as Cohens *f* for comparison of 3 groups (ED) and Cohens *d* for comparison of two groups (ICU). For better interpretation of non-significant findings 95% confidence intervals (CIs) are included to illustrate the range of plausible effect sizes. The estimated sample sizes required to achieve 80% power are shown in the last column.

| ***Emergency department population*** | | | | |
| --- | --- | --- | --- | --- |
|  | *Effect size (f)* | | | *n per group*  *at β=0.8* |
| **miR-10** | **0.25** | | | **52** |
| **miR-16** | **0.24** | | | **57** |
| **miR-21** | **0.35** | | | **35** |
|  | *Effect size (f)* | | *95% CI* | *n per group*  *at β=0.8* |
| miR-26 | 0.14 | | 0 – 0.27 | 165 |
| miR-27 | 0.10 | | 0 – 0.23 | 323 |
| miR-29 | 0.11 | | 0 – 0.24 | 267 |
| miR-93 | 0.13 | | 0 – 0.26 | 192 |
| miR-101 | 0.14 | | 0 – 0.26 | 165 |
| miR-127 | 0.08 | | 0 – 0.22 | 503 |
| miR-146 | 0.15 | | 0 – 0.28 | 144 |
| miR-192 | 0.14 | | 0 – 0.26 | 165 |
| miR-210 | 0.17 | | 0 – 0.29 | 113 |
| ***Intensive care population*** | | | | |
|  | *Effect size (d)* | | | *n per group*  *at β=0.8* |
| **miR-21** | **0.89** | | | **21** |
|  | *Effect size (d)* | *95% CI* | | *n per group*  *at β=0.8* |
| miR-10a | -0.07 | -0.66 – 0.52 | | 3205 |
| miR-16 | -0.57 | -1.17 – 0.03 | | 50 |
| miR-26 | 0.01 | -0.58 – 0.60 | | 156979 |
| miR-27 | -0.43 | -1.03 – 0.16 | | 86 |
| miR-29 | 0.08 | -0.50 – 0.67 | | 2454 |
| miR-93 | -0.26 | -0.85 – 0.33 | | 234 |
| miR-101 | -0.16 | -0.75 – 0.43 | | 615 |
| miR-127 | 0.00 | -0.71 – 0.71 | |  |
| miR-146 | 0.32 | -0.28 – 0.91 | | 155 |
| miR-192 | -0.09 | -0.68 – 0.50 | | 1939 |
| miR-210 | -0.02 | -0.61 - 0.57 | | 39246 |
